# Functional investigation of a QTL affecting resistance to *Haemonchus contortus* in sheep

**DOI:** 10.1186/1297-9716-45-68

**Published:** 2014-06-17

**Authors:** Guillaume Sallé, Carole Moreno, Simon Boitard, Julien Ruesche, Aurélie Tircazes-Secula, Frédéric Bouvier, Mathias Aletru, Jean-Louis Weisbecker, Françoise Prévot, Jean-Paul Bergeaud, Cathy Trumel, Christelle Grisez, Emmanuel Liénard, Philippe Jacquiet

**Affiliations:** 1 INRA, UMR1282, Infectiologie et Santé Publique, F-37380 Nouzilly, France; 2 Université François Rabelais de Tours, UMR1282 Infectiologie et Santé Publique, F-37000 Tours, France; 3 INRA, UR631, Station d'Amélioration Génétique des Animaux, BP 27, F-31326 Castanet-Tolosan, France; 4 INRA, UMR444, Laboratoire de Génétique Cellulaire, BP 27, F-31326 Castanet-Tolosan, France; 5 INRA, UE332, Domaine de la Sapinière, F-18390 Osmoy, France; 6 INRA, UE65, Domaine de Langlade, F-31450 Pompertuzat, France; 7 INRA, UMR1225, Interactions Hôtes – Agents Pathogènes (IHAP), F-31076 BP 87614, Toulouse, France; 8 Department of Clinical Sciences, INP-ENVT, Toulouse, France; 9 Université de Toulouse, Institut National Polytechnique (INP), Ecole Nationale Vétérinaire de Toulouse (ENVT), UMR1225, IHAP, Toulouse, France

## Abstract

This study reports a functional characterization of a limited segment (QTL) of sheep chromosome 12 associated with resistance to the abomasal nematode *Haemonchus contortus*. The first objective was to validate the identified QTL through the comparison of genetically susceptible (N) and resistant (R) sheep produced from Martinik × Romane back-cross sheep. The R and N genotype groups were then experimentally infected with 10 000 *H. contortus* larvae and measured for FEC (every three days from 18 to 30 days post-challenge), haematocrit, worm burden and fertility. Significant differences in FEC and haematocrit drop were found between R and N sheep. In addition, the female worms recovered from R sheep were less fecund. The second step of the characterization was to investigate functional mechanisms associated with the QTL, thanks to a gene expression analysis performed on the abomasal mucosa and the abomasal lymph node. The gene expression level of a candidate gene lying within the QTL region (PAPP-A2) was measured. In addition, putative interactions between the chromosome segment under study and the top ten differentially expressed genes between resistant MBB and susceptible RMN sheep highlighted in a previous microarray experiment were investigated. We found an induction of Th-2 related cytokine genes expression in the abomasal mucosa of R sheep. Down-regulation of the PAPP-A2 gene expression was observed between naïve and challenged sheep although no differential expression was recorded between challenged R and N sheep. The genotyping of this limited region should contribute to the ability to predict the intrinsic resistance level of sheep.

## Introduction

The failure of anthelmintic drugs is an issue of major concern throughout the world, especially for the control of small ruminants nematodes such as *Haemonchus contortus*[1]. Breeding animals with a better ability to resist infection by gastro-intestinal nematodes (GIN) has been proposed as an alternative strategy to drug treatment, and has already been implemented in Australia and New-Zealand [2,3].

This selection relies on the existing between-animal variation in the acquired immune response against GIN [4] which is mostly related, in murine models or in the sheep, to mounting an efficient Th-2 biased immune response driven by the IL4, IL5 and IL13 cytokines [5-7]. The humoral profile associated with this Th2 response involves IgA, IgG and IgE antibodies that control larval colonization, worm development and fecundity [5,8,9]. However, an innate component is also involved in the anti-nematode response. For instance, recent findings suggest that lectins contribute to entrapping worms in a mucus sheath, and would subsequently facilitate their elimination [10,11]. A previous microarray experiment comparing gene expression levels in resistant Martinik (MBB) and susceptible Romane (RMN) sheep infected by *H. contortus* found a stronger induction of Th2-related cytokines and also of lectin genes in MBB [12].

Numerous genetic mapping studies have identified regions of the genome explaining a non-negligible part of the inter-individual variation (known as Quantitative Trait Loci, QTL) in resistance to nematode infection [13-19]. The use of the ovine-specific DNA SNP chip showed that resistance to nematodes was determined by many genes with weak effect and some limited regions explaining a higher proportion of the genetic variation [17,19]. Candidate gene approaches have been carried out for the interferon gamma [20-22] and the major histocompatibility complex loci [23-26], although none of the other regions identified by genetic mapping strategies have been mined further. Identifying the mutations controlling ovine resistance to *H. contortus* should improve the ability to perform genetic selection by directly targeting the genes of interest through marker-assisted selection.

In a previous QTL mapping study for resistance to *Haemonchus contortus*, Sallé et al. found five QTL of greater interest on OAR5, 7, 12, 13 and 21 that affected Faecal Egg Count (FEC) and other parameters measured in a 1000 Martinik Black-Belly × Romane (MBB × RMN) back-cross (BC) lamb population [19]. A QTL region on OAR12, between 47 and 56 Mbp, explained 4% of observed variation in FEC at first and second infection and was detected in two different subsets of back-cross sheep and three independent sheep populations (Sarda*Lacaune [27], Merino [13] and in a free living Soay sheep population [14]). The purpose of this study was to perform a functional validation of this QTL region. Based on the within-family QTL detection results, some BC sheep were selected to produce BCxBC progeny that would carry two alleles associated with resistance or two alleles associated with susceptibility. BCxBC progenies were subsequently selected based on their genotypes for the investigated QTL and submitted to an exhaustive parasitological and haematological data collection. A gene expression study on the top ten differentially expressed genes between resistant MBB and susceptible RMN sheep highlighted in a previous microarray experiment [12] and on a candidate gene lying within the QTL region was also performed.

## Materials and methods

### Association between FEC at first infection and 4-SNP haplotype

Previous association analysis performed in a MBB × RMN BC flock (reported in [19]) identified a significant association between a 4-SNP haplotype (namely s39968, OAR12_62301297, OAR12_62347621, OAR12_62371899) located at 56.06 Mbp on OAR12 and FEC at first infection. Briefly, this analysis consisted in testing the effect of each 4-SNP haplotype window on the trait of interest, at every 0.05 Mbp. Haplotypes whose frequency was below 1% were discarded to limit standard error of the estimation. In addition, breed origin of the haplotype was taken into account, so that two identical-by-state (IBS) haplotypes were considered different if their breed origin was different.

**Figure 1:**
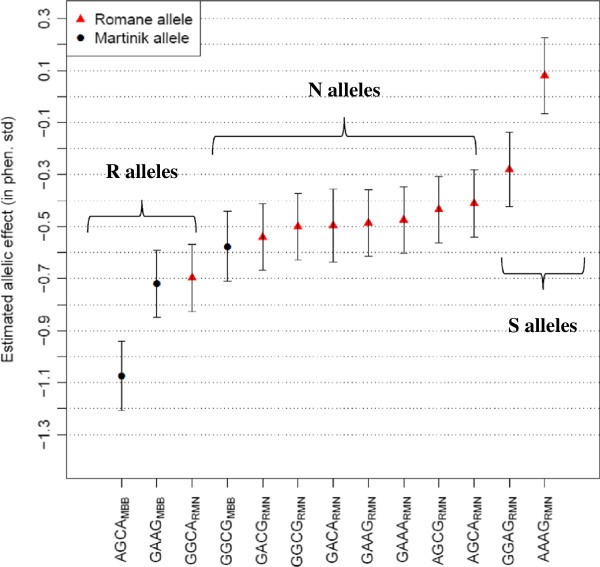
**Allelic effect of the 4-SNP haplotype estimated with the association analysis performed in the BC population.** The thirteen alleles of the 4SNP haplotype associated with Faecal Egg Count at first infection that were segregating in the back-cross population are plotted against their estimated effect (given in phenotypic standard deviation). Alleles inherited from the resistant Martinik breed are plotted in black and corresponding sequences are annotated with “MBB”, while alleles from the Romane breed are shown in red and annotated with ‘RMN”. R, N and S alleles stand for alleles being favourable, neutral and unfavourable toward *H. contortus* infection respectively.

From these results, two clusters of alleles with significant contrasted effects were defined (Figure 1, Additional files 1 and 2). A first group of two rare RMN alleles (GGAG_RMN_ and AAAG_RMN_), subsequently denoted S, showed the most unfavourable effects, *i.e.* associated to the highest FEC during *H. contortus* infection (Figure 1). In contrast to these S alleles, a cluster of three alleles (subsequently denoted R) was significantly more favourable toward limiting *H. contortus* infection. A difference of 0.58 σ_p_ was estimated between the S and the R alleles for FEC at first infection. The most favourable allele was segregating in the MBB breed (AGCA_MBB_) but one RMN allele (GGCA_RMN_) also belonged to this cluster supporting the resistance potential of this breed. Remaining alleles were considered as being neutral with respect to resistance for *H. contortus* infection (denoted N).

### Production of the R and N sheep

To investigate the biological properties of the identified QTL region, a marker-assisted mating of BC sheep was performed to produce lambs carrying particular combination of QTL alleles, i.e. RR, RN or NN. BC sheep were selected according to the QTL allele they carried. Chromosomes of every BC sheep were reconstructed using their 50 K SNP genotypes (described in [19]) and the LinkPHASE software [28], so that the QTL region could be traced from pure breed grand-parents to BC lambs. Two BC sires with RN genotype and one BC sire with NN genotype were selected for mating with 73 BC ewes (45 NN, 26 RN and two RR ewes). Because of their low frequency, the S alleles were not segregating in the remaining BC population. To randomize as much as possible the distribution of the other QTL in the BCxBC progenies, the three sires were mated to RN and NN ewes. The two RR ewes were mated to the RN sires to increase the number of RR genotypes. In the end 130 BCxBC sheep were born at the La Sapinière experimental farm (Osmoy, France).

### Sorting R and N sheep according to the association analysis

BCxBC sheep were genotyped with the 50 K ovine SNP chip (Illumina Inc, San Diego, CA, USA) and the same workflow as applied for their parents SNP data (described in [19]) was followed to select genotypes of interest. After data processing, 85 NN, 32 RN and 13 RR BCxBC progenies were counted. We retained the 54 sheep (22 NN, 20 RN and 12 RR) for which the chance of having inherited the QTL fragment from their ancestors was the highest. We finally compared the NN sheep (N group) against carriers of the R allele (R group, composed of the RN and RR sheep).

### Infection procedure, pathophysiological measurements and tissue sampling

Lambs were kept indoors from birth to the end of the experiment, thus remaining totally worm-free before their infection. Lambs were transferred from the experimental farm (La Sapinière farm, Osmoy France) where they were born to the experimental facilities (Langlade farm, Pompertuzat, France). Upon arrival in the experimental facilities, the selected BCxBC sheep were given a Vecoxan ND treatment (diclazuril, 1 mg/kg bodyweight, Janssen) at the recommended dose to prevent any coccidiosis outbreak. They were subsequently left indoors for a one-month acclimation period. After checking that no strongyle eggs were excreted, 44 sheep were infected orally with 10 000 infective L3 larvae of the *H. contortus* strain used in the previous QTL mapping study [19]. Ten additional uninfected lambs, i.e. five of each susceptibility group, were not challenged and reserved for the gene expression analysis to determine basal gene expression level within each susceptibility group. For practical purpose, control lambs were sacrificed two days after the challenge took place, whereas the 44 other infected animals were euthanized at 30 and 31 dpi. Euthanasia was performed by a veterinary surgeon with a lethal intra-venous injection of embutramide (T61, 6 mL/50 kg bodyweight, Intervet). In agreement with the current French regulations at the time of the experiment (2011), INRA procedures for the care of experiment animals were applied.

Intra-rectal collection of faeces was performed every three days from 18 days post infection (dpi) until 30 dpi for FEC counting following the McMaster method modified by Raynaud [29]. These traits were denoted FEC18, FEC21, FEC24, FEC27 and FEC30. Blood samples were collected just before infection, at 14 dpi and 27 dpi. Samples were processed by the Sysmex XT-2000iV haematology analyser (calibrated for sheep) hence providing a complete screening of haematological parameters. Reticulocytes (denoted RET_0_, RET_14_ and RET_27_ for samples taken before, at 14 and 27 dpi respectively) and white blood cell counts were obtained, i.e. lymphocytes (LYMPH_0_, LYMPH_14_ and LYMPH27), monocytes (MONO_0_, MONO_14_, and MONO27), neutrophils (NEUT_0_, NEUT_14_, NEUT_27_), eosinophils (EO_0_, EO_14_, EO_27_) and basophils (BASO_0_, BASO_14_ and BASO_27_). Haematocrit was also determined (denoted HCT_0_, HCT_14_ and HCT_27_).

Following euthanasia, abomasal (gastric) lymph nodes (ALN) and a patch of the abomasal fundic mucosa (AFM) were sampled and stored at -20°C in RNAlater (Ambion, USA). Abomasal contents and washings were collected and put into absolute alcohol. Worm burden (WB) was determined using 10% of the total abomasal content. The lengths of 35 intact adult female worms were determined and averaged (denoted FL) for each lamb. To determine the average number of eggs in utero, 20 female worms were digested individually into a bleaching mixture (40 mL of Milton agent diluted into 160 mL of distilled water) and 10% of the resulting mixture was sampled for eggs counting using an optical microscope (denoted FF). The average number of eggs in utero per female *H. contortus* was subsequently obtained by averaging over the twenty individual females. In addition, sheep were weighed before and at the end of the experimental challenge and the difference between the two measurements divided by 30 was considered as average daily gain (ADG).

### Gene expression measure

A gene expression comparison was performed between carriers of the GAAG_MBB_ allele (Ri group, *n* = 9) and carriers of the GAAG_RMN_ allele (Ni group, *n* = 8). These two particular alleles were chosen to be the most distinct in their effects and the most frequently distributed among the R and N sheep. Two groups of five uninfected control sheep (denoted Ru and Nu) were used to provide a basal gene expression level for the infected animals from the corresponding genotype.

To identify what genes underly the QTL, the annotated genes located within a 2-Mbp region centred at 56.06 Mbp on OAR12 were retrieved using the ovine genome website [30]. These genes are provided in Table 1. Given their known biological functions [31], we selected the PAPP-A2 gene for gene expression analysis as it was the only gene with obvious link to the immune response (Table 1).

**Table 1:** List of the genes lying within the QTL region under study

**Gene name**	**Position**	**Related GO functions**
RFWD2	55 199 014	DNA damage response
SGOL1	55 509 260	Cell cycle
PAPP-A2*	55 757 260	Regulation of Insulin-like Growth Factor (IGF) activity
ASTN1	56 095 325	Nervous system development
FAM5B	56 545 767	Cell cycle, nervous system development
TAF9	56 864 943	Initiation of transcription by RNA polymerase II

Annotated genes comprised within the 55.1 and 57.1 Mbp interval of OAR12 are provided. Their related gene ontology function has been retrieved from the NCBI’s website [31]. Position is given in bp and corresponds to the starting position as indicated on the ovine sheep genome website [30]. PAPP-A2*: as this gene was the only one within the QTL region with an obvious role in immunity, only this gene was examined in gene expression analysis.

Additionally, a previous microarray study [12] found that, some genes (Table 2) were differentially expressed between infected MBB and RMN sheep, either in AFM (LGALS15, ITLN2, TFF3) or in ALN (TNFRSF4, CCL26, CXCL14) or both (IL4, IL5, IL13, TNFα, IFNγ). We thus performed a gene expression analysis on these candidates to assess whether they would be correlated to the QTL region under study.

**Table 2:** List of the genes considered for gene expression analysis

		**Gene name**	**OAR**	**Position**	**Related GO functions**	**Tissue in which the gene was tested**
Within QTL		PAPP-A2	12	55 757 260	Regulation of Insulin-like Growth Factor (IGF) activity	AFM, ALN
	Innate response	LGALS15	-	-	Carbohydrate binding	AFM
		ITLN2	1	110 480 137	Response to nematode	AFM
		TFF3	1	260 633 891	Molecular function	AFM
		IL4	5	19 237 699	Cytokine activity	AFM, ALN
		IL5	5	19 436 357	Cytokine activity	AFM, ALN
		IL13	5	19 261 902	Cytokine activity	AFM, ALN
Outside QTL	Acquired response	TNFα	20	26 854 381	Cytokine activity	AFM, ALN
		IFNγ	3	151 528 005	Cytokine activity	AFM, ALN
		TNFRSF4	12	49 359 903	Tumor necrosis factor-activated receptor activity	ALN
		CCL26	24	33 952 859	Chemokine (C-C motif) ligand 26	ALN
		CXCL14	5	44 432 648	Chemokine (C-X-C motif) ligand 14	ALN

The genes whose expression level has been measured are listed with their respective gene name, chromosome (OAR) and starting position (given in bp) and gene ontology function as provided by the NCBI’s website [31]. Expression levels were measured in the abomasal fundic mucosa (AFM)or in the abomasal lymph node (ALN) or both.

Total mRNA from abomasal fundic mucosa (AFM) and draining lymph nodes (ALN) of the 27 R and N sheep was extracted following the commercial RNeasy Mini Kit (Qiagen). The quality of the recovered RNA was monitored by A260/A280 spectrophotometry. RNAs were subsequently reverse-transcribed to cDNA with the Reverse Transcriptase commercial kit (Invitrogen).

Primers were designed for expression analysis using the “primer-BLAST” NCBI website [32]. Secondary structures were identified with the Mfold website [33] and selected primer sequences were blasted against the third version of the ovine genome to ensure specificity of their target. The qPCR was performed with three replicates per sample. Gene expression levels of five reference genes, namely *β-ACTIN*, *TYQ*, *SDH*, *S26Q* and *HPRT*, were measured. Their respective gene-wise stability values were estimated as reported in [34] and most stable genes were kept for subsequent analyses.

### Statistical analyses

Strong departures from normality were found for FEC data (using the UNIVARIATE procedure implemented in the SAS software, SAS 2001, Cary NC) that were corrected by a 4^th^ root transformation.

Resistance to nematodes is known to be polygenic and other QTL did segregate in this population [19]. Therefore, a genomic value (gEBV) evaluating the effect of the genomic background on FEC at first infection was estimated for each BCxBC sheep. FEC was considered as a proxy for resistance to nematodes in order to maximize the data available for estimating the gEBV by using the whole set of BC and BCxBC data (*n* = 1200 records). The computation was performed with the Bayes C genomic selection method [35] implemented in the GS3 software [36]. FEC was modelled as the sum of a mean, the already identified environmental fixed effects [19], i.e. sex, age at sampling and litter size, the marker effect and a residual term. None of the markers located on OAR12, i.e. the chromosome harbouring the QTL region, were included in the analysis as it was expected to partially sweep the genetic variance explained by the QTL into the gEBV as reported elsewhere [37].

The PROC MIXED procedure implemented in the SAS software [38] was used to test for significant differences between R and N genotypic groups for each of the measured parasitological and haematological data. Usually encountered environmental effects were considered as fixed effects and the computed gEBV was fitted to the model as a covariate to account for the effect of the rest of the genome. For haematological parameters, basal value of the considered parameter (indexed by 0) was considered as covariate to account for potential inter-individual variation before the beginning of the experiment.

Normality of the cycle time (C_T_) values distribution was checked using the Shapiro-Wilk test implemented in the R software [39]. One outlier from the Ri group (sheep 12493, see Additional file 3), with C_T_ values for house-keeping genes exceeding a ± three standard deviations range, was discarded from the AFM dataset. Differential expression was tested following the 2^-ΔΔC^_T_ method [40]. For each individual the mean C_T_ value from the three replicates was expressed as a relative abundance to the average expression level from the reference genes, denoted ΔC_T_.

To assess any modifications before or after infection related to the QTL genotype, pair-wise comparisons between Ru and Nu and Ri and Ni groups respectively were tested. To assess whether any differences between Ri and Ni groups was due to a lack of change between the infected sheep and their naïve counterparts, the Ri to Ru and Ni to Nu comparisons were tested.

Pair-wise comparisons were computed as the difference between ΔC_T_ of the two groups as ΔΔC_T_ = ΔC_T1_ – ΔC_T2_. For comparing Ri and Ni gene expression levels, ΔC_T_ of the infected sheep was expressed as a relative abundance to the average expression of the corresponding susceptibility control group and the subsequent ΔΔC_T_ value computed as:

$$\Delta \Delta C_{T}=\left(\Delta C_{\text{TRi}}‒\text{mean}\left(\Delta C_{\text{TRu}}\right)\right)-\left(\Delta C_{\text{TNi}}‒\text{mean}\left(\Delta C_{\text{TNu}}\right)\right)$$

This correction was applied to correct for putative differences in gene expression levels between R_U_ and N_U_ sheep before infection took place.

Fold change in gene expression between considered groups was computed as 2^-ΔΔC^_T_[40]. Subsequently, a Wilcoxon test was applied to determine any significant difference between the compared groups. To account for multiple testing, a nominal *p*-value of 1% was considered for significance leading to less than one significant difference occurring by chance. An additional suggestive threshold was considered for nominal *p*-value below 5%. The complete data processing was performed using an in-house R script [39].

## Results

### Phenotypic comparison of the R and N sheep

The average gEBV of the R and N groups were equivalent (*p* = 0.34) hence allowing a standardized comparison of their QTL genotype.

A selection of parasitological and haematological data for the two groups is provided in Table 3 (the complete list of recorded traits and associated statistics are provided in Additional file 4). The 4-SNP-based clustering of the BCxBC sheep allowed prediction of true “high-” and “low-FEC” sheep, as illustrated by the 601 eggs/g and 11546 eggs/g difference obtained between the R and N groups at 18 and 30 dpi (*p* = 0.02 and 0.01 respectively in both cases, Table 3). Further, the 4-SNP genotype was associated with strong differences in the length and fertility of female worms (Table 3). Female *H. contortus* collected from R sheep were 1.6 mm shorter on average (*p* = 0.0013) and showed 1.5 times fewer eggs in utero (*p* = 5.10^-4^) than those recovered in N sheep (525 and 358 eggs in utero/female for the N and R groups respectively, Table 3). These parasitological findings also correlated with the reduced blood loss at 14 and 27 dpi in R lambs (*p* = 0.03), and a higher production of reticulocytes in the N lambs (Table 3). However no significant differences were observed for WB between the two groups (*p* = 0.73). From a production perspective, no differences of growth rates could be found between the two groups (*p* = 0.23).

**Table 3:** Phenotypic comparison of R and N sheep

**Trait**	**R**		**N**		***p*-value**	**Phenotypic difference (σ**_**p**_**)**
	**Mean**	**Std**	**Mean**	**Std**		
FEC18 (eggs/g)	126	289	727	1153	0.02	-0.70
FEC21 (eggs/g)	1160	1813	3865	3705	0.06	-0.54
FEC24 (eggs/g)	3874	3031	6968	5233	0.19	-0.37
FEC27 (eggs/g)	5798	4075	9150	6422	0.11	-0.44
FEC30 (eggs/g)	13213	8286	24759	17730	0.01	-0.70
WB (no. worms)	4084	1885	4395	1267	0.73	0.10
FL (in mm)	18.7	1.4	20.3	1.6	0.0013	-0.95
FF (no. eggs in utero)	358	112	525	182	0.0005	-1.03
HCT14 (%)	34.2	3.2	31.9	3.5	0.02	0.53
HCT27 (%)	30.3	3.4	27.5	3.3	0.01	0.65
RET27 (%)	0.173	0.200	0.816	1.009	0.01	-0.77
ADG (kg/day)	0.109	0.045	0.137	0.049	0.23	-0.59

Raw parasitological data and haematological profiles of the R and N groups are reported in this Table. *P*-values were computed after correction for fixed effect and statistical transformation when appropriate. Phenotypic differences are the difference between the R and the N groups expressed in phenotypic standard deviation (σ_p_).

### Testing for differential gene expression

**Figure 2:**
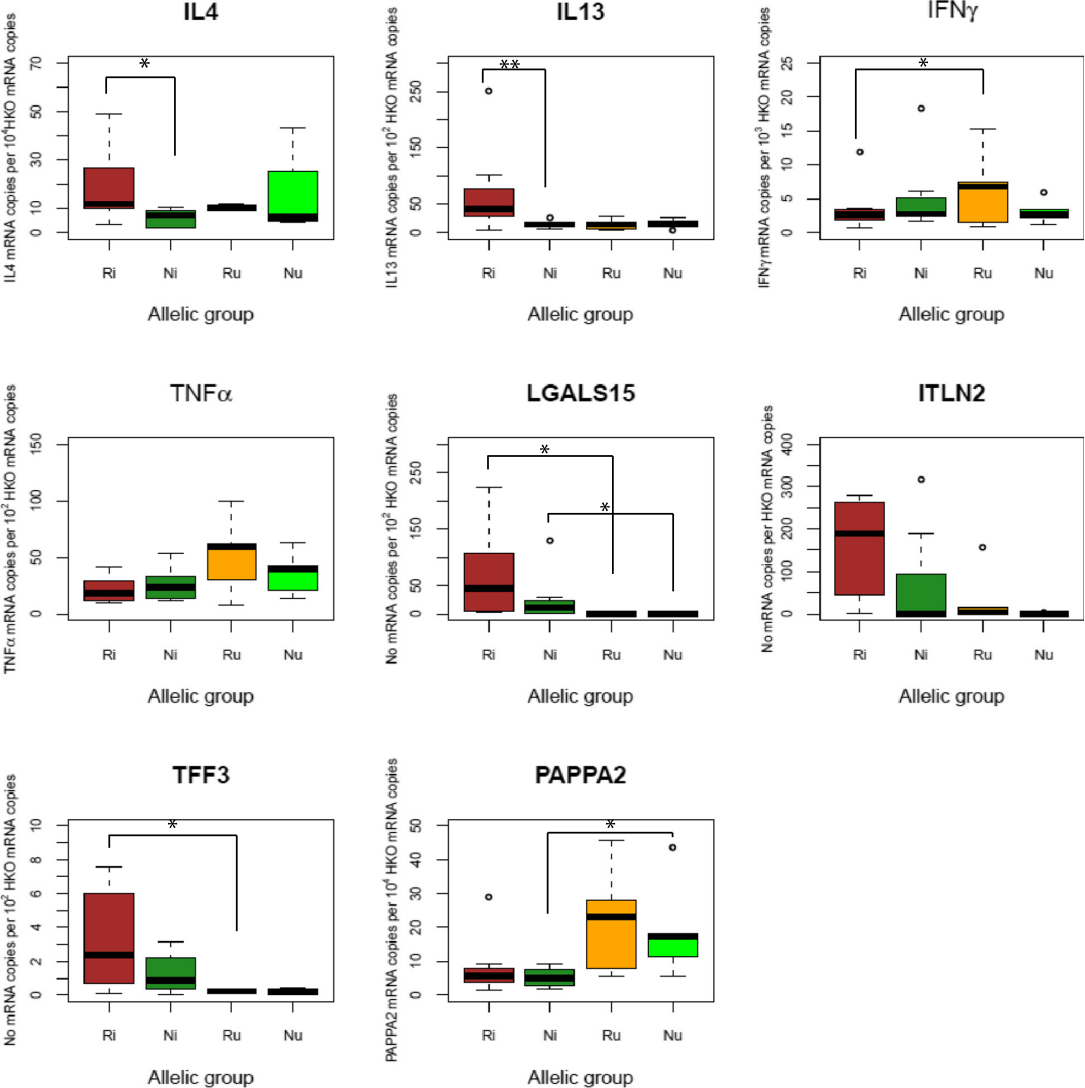
**Gene expression level for a subset of candidate genes in the abomasal mucosa of each allelic group 30 days post-infection by *H. contortus.*** A boxplot of the gene expression levels is provided for each of the considered allelic group. The horizontal bar within the box is the median value while open circles indicate values falling above the upper (or below the lower) quartile plus (minus) 1.5 times the interquartile distance. Ri and Ni are the infected resistant and susceptible lambs while Ru and Nu are the non-infected control resistant and susceptible lambs. Asterisks indicate suggestive (*p* < 0.05, shown as “*”) or significant (*p* < 0.01, shown as “**”) differences between groups.

**Figure 3:**
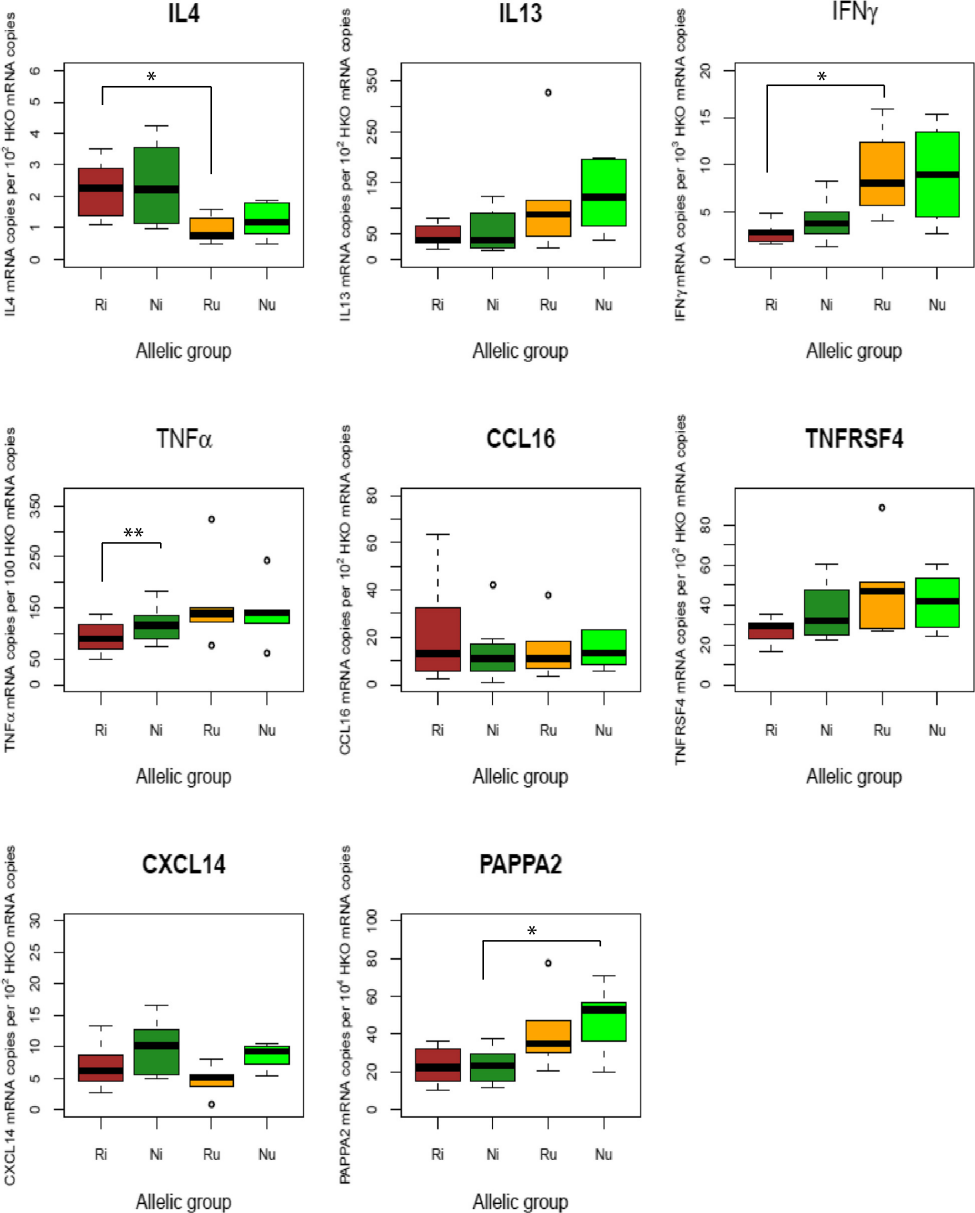
**Gene expression level for a subset of candidate genes in the abomasal lymph node of each allelic group 30 days post-infection by *H. contortus.*** A boxplot of the gene expression levels is provided for each of the considered allelic group. The horizontal bar within the box is the median value while open circles indicate values falling above the upper (or below the lower) quartile plus (minus) 1.5 times the interquartile distance. Ri and Ni are the infected resistant and susceptible lambs while Ru and Nu are the non-infected control resistant and susceptible lambs. Asterisks indicate suggestive (*p* < 0.05, shown as “*”) or significant (*p* < 0.01, shown as “**”) differences between groups.

The gene expression profiles of each Ri and Ni as well as their respective control, Ru and Nu, are shown in Table 4, Figures 2 and 3. C_T_ values for each measured gene are provided in Additional file 3.

**Table 4:** Differential expression of the selected gene set in abomasal mucosa (AFM) and draining lymph node (ALN)

**Tissue**	**Gene**	**FC Ri vs Ni**	**FC Ri vs Ru**	**FC Ni vs Nu**	**FC Ru vs Nu**
AFM	IL4	4.07*	2.22	0.54	1.16
	IL13	4.51**	4.79*	1.06	0.93
	IFNγ	0.83	1.13	1.36	1.47
	TNFα	0.85	0.63	0.74	1.23
	LGALS15	7.02	576*	82.0*	0.60
	ITLN2	6.97	22.4	3.22	4.94
	TFF3	1.61	6.86*	4.26	1.50
	CCL26	Not tested in this tissue			
	TNFRSF4	Not tested in this tissue			
	CXCL14	Not tested in this tissue			
	PAPPA2	1.18	0.34	0.29*	1.08
ALN	IL4	1.22	2.33*	1.91	0.81
	IL13	1.24	0.50	0.41	0.78
	IFNγ	0.69	0.33**	0.48	1.08
	TNFα	0.70*	0.62	0.88	1.12
	LGALS15	Not tested in this tissue			
	ITLN2	Not tested in this tissue			
	TFF3	Not tested in this tissue			
	CCL26	1.65	1.13	0.69	0.87
	TNFRSF4	0.68	0.59	0.87	1.11
	CXCL14	1.45	1.56	1.08	0.44
	PAPPA2	1.06	0.52	0.49*	0.88

Fold changes (FC) between the four considered groups (resistant allele carriers infected and uninfected (Ri and Ru respectively) and susceptible allele carriers infected and uninfected (Ni and Nu respectively) are provided. Significant differences (nominal *p*-value < 0.01) are indicated with **, and suggestive differences (nominal *p*-value < 0.05) are marked as *.

Among the annotated genes retrieved within a 2 Mbp region centred at 56.06 Mbp (Table 1), the pappalysin gene (PAPP-A2) was identified as a putative candidate due to its regulatory effect on IGF-1. This gene was found down-regulated in the Ni in comparison to their uninfected counterparts in both AFM and ALN (*p* = 0.02 and 0.05 respectively). However the similar down-regulation trend observed between Ri and Ru sheep was not significant (Table 4, Figures 2 and 3). Neither was the PAPP-A2 expression differential between Ri and Ni groups (Table 4, Figures 2 and 3).

Ru and Nu sheep gene expression levels were compared to test for differences of the basal expression pattern linked to the QTL genotype. However no significant nor suggestive differences in gene expression levels was recorded between the Ru and Nu sheep.

Similarly, Ri and Ni sheep exhibited similar expression levels of AFM-specific genes involved in the innate response, i.e. lectin genes (LGALS15 and ITLN2) and the TFF3 gene (Table 4). A significant induction of LGALS15 mediated by *H. contortus* infection was observed between infected groups and their respective controls (*p* = 0.04 between the Ri and Ru groups and *p* = 0.02 between the Ni and Nu sheep, Table 4 and Figure 2).

Both suggestive and significant variations were observed between Ri and Ni sheep for some components of the acquired response (Table 4). Indeed, Ri sheep demonstrated a 4-fold increase in the IL-4 and IL-13 gene expression level in AFM in comparison to the Ni sheep (*p* = 0.04 and *p* = 0.02 for IL4 and IL13 respectively, Table 4 and Figure 2). The difference of IL13 expression between Ri and Ni seemed to be related to the lack of IL13 induction in Ni sheep as no significant difference was observed between Ni and Nu sheep (*p* = 0.72). Also, a slight suggestive down-regulation of TNFα was observed between Ri and Ni sheep (Table 4, Figure 3). The apparent stronger Th2 cytokine environment in the Ri sheep was also reinforced by a significant reduction by a 1/3 factor of the IFNγ expression (*p* = 0.01) in ALN between Ri and Ru sheep which could not be found between Ni and Nu sheep (Table 4, Figure 3). In addition, a non-significant down-regulation of TNFRSF4 was also observed between the same groups (Table 4, Figure 3).

## Discussion

The reported study aimed at mining the functional properties of a QTL region associated with resistance to *H. contortus*. The first objective was to validate the identified QTL through the comparison of individuals selected on their particular QTL alleles. The second related goal was to investigate functional mechanisms associated with the QTL, thanks to a wider range of phenotypes, including gene expression analysis. Abundant literature has been produced on the role of particular loci like the MHC [24-26,41,42] or IFNγ [20,22]. Other research teams mined the functional differences between divergent lines of sheep selected for low or high FEC [43-46], but to our knowledge, this is the first attempt to functionally investigate the properties of a positional candidate without prior evidence of a functional candidate affecting resistance to GIN in sheep.

Our results demonstrated that a 4-SNP haplotype of OAR12 could discriminate between resistant and susceptible lambs. The genotypic groups based on this 4-SNP genotype showed significant reduction in FEC (ranging between 600 and 11 000 eggs/g difference between R and N groups) haematocrit drop and worm fecundity (1.5 times less eggs in utero in *H. contortus* females in the R sheep). Given that the two genotypic groups had identical genomic background, our findings provide strong functional support for the QTL signal detected by the mapping approach.

As a wider RNAseq experiment is to be undertaken, only a limited set of targeted genes were measured for gene expression as a primary screening. These genes were either directly lying within the QTL region under study, or had been previously involved as major candidates in the resistance difference between MBB and RMN breeds.

The nearest annotated gene underlying the 4SNP region was the PAPP-A2 gene. It encodes a protease cleaving Insulin Growth Factor Binding Protein-4 (IGFBP4), therefore increasing the bioavailability of the Insulin Growth Factor (IGF) [47]. The IGF gene plays a role in the immune response [47-49] and was recently highlighted as a key player during wound healing associated with a nematode-induced Th2 response in a murine model [50]. In addition, PAPP-A2 expression is highly dependent of pro-inflammatory cytokines such as IFNγ and TNFα [47,48] and it is induced during wound healing [47]. Interestingly, PAPP-A2 gene expression was significantly reduced in infected R and N sheep, between Nu and Ni (*p* = 0.02 and *p* = 0.05 in AFM and ALN respectively) although the reduction was not significant between Ri and Ru sheep (*p* = 0.09 and *p* = 0.07 in AFM and ALN respectively). To date, this is the first report of a relationship between PAPP-A2 and *H. contortus* infection. However no differences were observed between resistant and susceptible sheep after infection, and expression levels of PAPP-A2 did not follow the trends of other cytokines known to have regulatory effects, such as IL4 (up-regulation), IFNγ (down-regulation) or TNFα (up-regulation). Therefore the putative role of this gene still remains to be confirmed. Further, the observed gene expression levels might reflect some process that occurred earlier during the infection process preventing identification of differences between the different susceptibility groups. In particular, it would be interesting to examine IGF-1 and associated PAPP-A2 expression levels early in the infection process and as early as four dpi as reported for another murine model [50].

A previous microarray experiment identified a few genes differentially expressed between pure breed MBB and RMN sheep. Expression of these genes was measured to identify any relationship with the QTL genotype. From the expression data, no particular relationship could be drawn between this QTL and components of the innate response, such as lectins or TFF3. Such a relationship, if any, might occur at a different time point from the anti-*H. contortus* response. The only similar expression variation to the array study was the significant induction of LGALSL15 between naïve and infected sheep [12].

On the contrary, the observed cytokine gene expression ratios suggest an effect on worm fecundity through the mounting of a stronger Th-2 type environment as illustrated by the increase in IL4 and IL13 expression [5,46]. Further, the IFNγ expression, known to be associated with susceptibility to GIN in murine models [5] was also down-regulated in Ri sheep in comparison to the Ru sheep. The additional slight down-regulation of TNFRSF4 (*p* = 0.07) between the same two groups constitutes another factor contributing to the mounting of a Th-2 environment against GIN while repressing the Th-1 response. Indeed the inhibition of the TNFRSF4 cytokine is known to induce a more efficient expulsion of helminths in mice models of nematode infection [51,52]. Same findings of a stronger Th-2 environment were already reported while comparing pure breed Martinik and Romane sheep either at 4 and 30 dpi [53] or at 8 dpi [12]. IL5 is an additional Th-2 associated cytokine that was also found to be over-expressed in pure breed Martinik infected by *H. contortus*[12,53] and in resistant Blackface sheep facing *T. circumcincta* infection [46]. In our experiment however, IL5 expression level was undetected after 40 qPCR cycles in 88% of the replicates, either using the primers published in [53] or after designing new primers (data not shown).

Proposing a detailed mode of action of the investigated QTL region would require more detailed investigation. However, extreme QTL alleles showed great contrasts in their effect on FEC, sheep blood loss and female worm fecundity. Since every lamb was inoculated with a similar infecting dose and no differences in worm burden could be found, the strong differences in female fertility reported here cannot be related to reduction in fecundity associated with density dependent effects [9,54]. Hence, observed differences in worm fecundity seem to be directly mediated by the QTL under study. However, the QTL allelic group explained 26% of the variation for this trait at most, suggesting other factors are also involved as reported elsewhere [9,54]. Further, significant differences in haematocrit were consistently observed between R and N groups at 14 and 27 dpi. Provided reticulocyte production was significantly higher in susceptible lambs after infection only, observed differences reflected a difference in true blood loss and not a higher regeneration ability of the resistant lambs. In addition, haematocrit at 27 dpi was negatively correlated to worm size (-0.43) and worm fecundity (-0.53). Both findings lead to the hypothesis that the investigated QTL region could limit worm feeding hence reducing their growth (shorter females) and fecundity (lower eggs recovered in utero). This could be mediated by the stronger Th-2 response that seems to be mounted by R sheep after infection. Indeed, local IgA response limits worm fecundity in *T. circumcincta* infection [9,55] and in *H. contortus* infection [7]. More recent findings also support the relationship between T-cells number and worm female length [56]. Additional investigations on the characterization of subpopulations of T-cells in allelic carriers of each type could bring additional insights. As well, histological examination of abomasal mucosa of extreme animals could also confirm the stronger Th-2 response by measuring the eosinophilic infiltration and the number of mast cells. Additional detrimental factors like an increase of lectins in mucus could not be demonstrated in this study.

An additional meta-analysis of other parasite infection datasets gathered in the European 3SR project [57] is currently in process to confirm this region as a key player across multiple European breed, and eventually refine its location [58]. However, the back-cross design will not permit further refinement of the causative mutation explaining these variations. Therefore, pure breed association analyses are currently in progress to help refine the QTL position and a RNAseq experiment will be undertaken to widen the scope of functional candidates.

## Competing interests

The authors declare that they have no competing interests.

## Authors’ contributions

GS designed the experiment, sampled animals, performed parasitological and haematological measurements and molecular biology, analysed data and drafted the manuscript. CM and PJ designed the experiment, sampled animals and were the grant holders. SB performed the hard selection signatures analyses. JR supervised the experiment and participated in animal samplings. MA and JL took care of the animals and FB carried out the sheep mating. AT, FP and CG sampled animals and performed molecular biology. CT performed haematological analyses. JP sampled animals and performed parasitological measures. EL sampled animals and analysed the data. All authors read and approved the final manuscript.

## Supplementary Material

Additional file 1 **Frequency and estimated effects of the 4-SNP haplotype associated to Faecal Egg Count at first infection in the back-cross population.** The estimated effects of the 4-SNP haplotypes identified in the back-cross population are provided in this file and given in phenotypic standard deviation. Frequencies of each allele are reported for both the back-cross population and the BCxBC progeny. a: AGCA_MBB_, AGCA allele from the Martinik Black-Belly breed; GGCA_RMN_, GGCA allele from the Romane breed; b: allelic effect is given in phenotypic standard deviation; standard errors of the estimates are indicated in brackets. Click here for file

Additional file 2 **Pair-wise comparison of the effects for every allele identified in the back-cross population.** For each couple of allele, a t-test has been applied considering respective estimated allelic effects and associated standard error from the QTL detection analysis and inferring the number of observations from the allelic frequency. The associated *p*-values are reported in the Table. A *p*-value below 0.05 (in bold) was considered as significant. The two clusters of alleles with significant contrasted effects are in italic. The MBB subscript indicates alleles inherited from the resistant Martinik breed (all other alleles segregated in the susceptible Romane breed). Click here for file

Additional file 3 **Geometric mean and associated standard deviation of measured Ct values in abomasal fundic mucosa (A) and abomasal lymph node (B) for each sheep × gene combination.** The geometric means and associated standard deviations of the measured C_T_ values for the three replicates are given for every sheep × gene combination. The five housekeeping genes were also provided. Any Ct value above 40 cycles were not considered for analyses; NA values for s indicates that either no or one Ct value was retained for analysis hence making it impossible to compute the mean or the standard deviation or both. Ri and Ru are respectively experimentally infected and uninfected resistant sheep while Ni and Nu are the genetically susceptible counterparts. A. Geometric mean (μ) and associated standard deviation (s) of measured Ct values for each sheep × gene combination in abomasal fundic mucosa (AFM). Any Ct value above 40 was discarded; NA values for s indicates that either no or one Ct value was retained for analysis. Ri and Ru are respectively experimentally infected and uninfected resistant sheep while Ni and Nu are the genetically susceptible counterparts. B. Geometric mean (μ) and associated standard deviation (s) of measured Ct values for each sheep × gene combination in abomasal lymph node (ALN). Any Ct value above 40 was discarded; NA values for s indicates that either no or one Ct value was retained for analysis. Ri and Ru are respectively experimentally infected and uninfected resistant sheep while Ni and Nu are the genetically susceptible counterparts. Click here for file

Additional file 4 **List of parasitological and haematological traits in the R and N sheep.** The average performance of the R and N sheep is provided for every single trait monitored during experimental challenge by *H. contortus* and after necropsy. Average raw data are provided for the ease of reading, while statistical tests were performed after correction for fixed effect (e.g. sheep sex) and statistical transformation when appropriate. Click here for file
