# Supplementary material for: Functional investigation of a QTL affecting resistance to Haemonchus contortus in sheep
Source: Vet Res. 2014 Jun 17;45(1):68. doi: 10.1186/1297-9716-45-68 (PMC4077151; doi:10.1186/1297-9716-45-68)
Supplement: Additional file 1 — Frequency and estimated effects of the 4-SNP haplotype associated to Faecal Egg Count at first infection in the back-cross population. The estimated effects of the 4-SNP haplotypes identified in the back-cross population are provided in this file and given in phenotypic standard deviation. Frequencies of each allele are reported for both the back-cross population and the BCxBC progeny. a: AGCAMBB, AGCA allele from the Martinik Black-Belly breed; GGCARMN, GGCA allele from the Romane breed; b: allelic effect is given in phenotypic standard deviation; standard errors of the estimates are indicated in brackets. [file 1297-9716-45-68-S1.doc]

| 4-SNP allele | Frequency in the BC population | Allelic effect estimated in the BC population | Frequency in the BCxBC population | | Note | | | | |
| --- | --- | --- | --- | --- | --- | --- | --- | --- | --- |
|  |  |  | Infected | Control |
| AGCA_MBB_ | 0.25 | -1.07 (0.13) | 0.14 | 0.21 | Alleles used for  predicting the most resistant BCxBC sheep | | | | |
| GAAG_MBB_ | 0.5 | -0.72 (0.13) | 0.05 | 0.12 |
| GGCA_RMN_ | 0.1 | -0.7 (0.13) | 0.02 | 0 |
| GGCG_MBB_ | 0.25 | -0.58 (0.13) | 0.06 | 0 |  |  | |  |  |
| GACG_RMN_ | 0.19 | -0.54 (0.13) | 0.23 | 0 |  |  | |  |  |
| GGCG_RMN_ | 0.15 | -0.5 (0.13) | 0.11 | 0.44 |  |  | |  |  |
| GACA_RMN_ | 0.02 | -0.5 (0.13) | 0 | 0 |  |  | |  |  |
| GAAG_RMN_ | 0.15 | -0.49 (0.13) | 0.09 | 0.12 | Alleles regarded as neutral toward | | | | |
| GAAA_RMN_ | 0.12 | -0.47 (0.13) | 0.03 | 0.09 | *H. contortus* infection | | | | |
| AGCG_RMN_ | 0.14 | -0.43 (0.13) | 0.03 | 0 |  |  |  | |  |
| AGCA_RMN_ | 0.09 | -0.41 (0.13) | 0.24 | 0.03 |  |  |  | |  |
| GGAG_RMN_ | 0.02 | -0.28 (0.13) | 0 | 0 | Alleles associated to the most unfavourable effect toward *Hcontortus* infection in the BC population | | | | |
| AAAG_RMN_ | 0.01 | 0.08 (0.13) | 0 | 0 |
