# Supplementary material for: Functional investigation of a QTL affecting resistance to Haemonchus contortus in sheep
Source: Vet Res. 2014 Jun 17;45(1):68. doi: 10.1186/1297-9716-45-68 (PMC4077151; doi:10.1186/1297-9716-45-68)
Supplement: Additional file 2 — Pair-wise comparison of the effects for every allele identified in the back-cross population. For each couple of allele, a t-test has been applied considering respective estimated allelic effects and associated standard error from the QTL detection analysis and inferring the number of observations from the allelic frequency. The associated p-values are reported in the Table. A p-value below 0.05 (in bold) was considered as significant. The two clusters of alleles with significant contrasted effects are in italic. The MBB subscript indicates alleles inherited from the resistant Martinik breed (all other alleles segregated in the susceptible Romane breed). [file 1297-9716-45-68-S2.doc]

|  | GAAG_MBB_ | GGCA | GGCG_MBB_ | GACG | GGCG | GACA | GAAG | GAAA | AGCG | AGCA | GGAG | AAAG |
| --- | --- | --- | --- | --- | --- | --- | --- | --- | --- | --- | --- | --- |
| AGCA_MBB_ | 0.0602 | 0.0427 | 0.0084 | 0.0042 | 0.0022 | 0.0052 | 0.0017 | 0.0014 | 0.0007 | 0.0005 | 0.0003 | <0.0001 |
| GAAG_MBB_ |  | 0.8963 | 0.4410 | 0.3285 | 0.2327 | 0.2525 | 0.2083 | 0.1861 | 0.1239 | 0.0960 | 0.0310 | 0.0008 |
| GGCA |  |  | 0.5151 | 0.3904 | 0.2811 | 0.3003 | 0.2529 | 0.2269 | 0.1537 | 0.1201 | 0.0393 | 0.0011 |
| GGCG_MBB_ |  |  |  | 0.8450 | 0.6798 | 0.6803 | 0.6328 | 0.5873 | 0.4474 | 0.3741 | 0.1395 | 0.0041 |
| GACG |  |  |  |  | 0.8254 | 0.8181 | 0.7743 | 0.7242 | 0.5664 | 0.4809 | 0.1870 | 0.0057 |
| GGCG |  |  |  |  |  | 0.9833 | 0.9472 | 0.8947 | 0.7242 | 0.6278 | 0.2624 | 0.0087 |
| GACA |  |  |  |  |  |  | 0.9666 | 0.9167 | 0.7539 | 0.6612 | 0.2959 | 0.0143 |
| GAAG |  |  |  |  |  |  |  | 0.9472 | 0.7743 | 0.6754 | 0.2888 | 0.0099 |
| GAAA |  |  |  |  |  |  |  |  | 0.8255 | 0.7244 | 0.3172 | 0.0114 |
| AGCG |  |  |  |  |  |  |  |  |  | 0.8948 | 0.4245 | 0.0172 |
| AGCA |  |  |  |  |  |  |  |  |  |  | 0.4988 | 0.0224 |
| GGAG |  |  |  |  |  |  |  |  |  |  |  | 0.1025 |
