# Supplementary material for: Functional investigation of a QTL affecting resistance to Haemonchus contortus in sheep
Source: Vet Res. 2014 Jun 17;45(1):68. doi: 10.1186/1297-9716-45-68 (PMC4077151; doi:10.1186/1297-9716-45-68)
Supplement: Additional file 3 — Geometric mean and associated standard deviation of measured Ct values in abomasal fundic mucosa (A) and abomasal lymph node (B) for each sheep × gene combination. The geometric means and associated standard deviations of the measured CT values for the three replicates are given for every sheep × gene combination. The five housekeeping genes were also provided. Any Ct value above 40 cycles were not considered for analyses; NA values for s indicates that either no or one Ct value was retained for analysis hence making it impossible to compute the mean or the standard deviation or both. Ri and Ru are respectively experimentally infected and uninfected resistant sheep while Ni and Nu are the genetically susceptible counterparts. A. Geometric mean (μ) and associated standard deviation (s) of measured Ct values for each sheep × gene combination in abomasal fundic mucosa (AFM). Any Ct value above 40 was discarded; NA values for s indicates that either no or one Ct value was retained for analysis. Ri and Ru are respectively experimentally infected and uninfected resistant sheep while Ni and Nu are the genetically susceptible counterparts. B. Geometric mean (μ) and associated standard deviation (s) of measured Ct values for each sheep × gene combination in abomasal lymph node (ALN). Any Ct value above 40 was discarded; NA values for s indicates that either no or one Ct value was retained for analysis. Ri and Ru are respectively experimentally infected and uninfected resistant sheep while Ni and Nu are the genetically susceptible counterparts. [file 1297-9716-45-68-S3.doc]

**A Geometric mean (µ) and associated standard deviation (s) of measured Ct values for each sheep x gene combination in abomasal fundic mucosa (AFM).**

| Sheep | Group | Tissue | House-Keeping Genes | | | | | | | | | | Genes of interest | | | | | | | | | | | | | | | |
| --- | --- | --- | --- | --- | --- | --- | --- | --- | --- | --- | --- | --- | --- | --- | --- | --- | --- | --- | --- | --- | --- | --- | --- | --- | --- | --- | --- | --- |
|  |  |  | HPRT | | S26Q | | SDH | | TYQ | | BACTIN | | IL4 | | IL13 | | IFNγ | | TNFα | | LGALSL15 | | ITLN2 | | TFF3 | | PAPPA2 | |
|  |  |  | μ | σ | μ | σ | μ | σ | μ | σ | μ | σ | μ | σ | μ | σ | μ | σ | μ | σ | μ | σ | μ | σ | μ | σ | μ | σ |
| 12402 | Ru | AFM | 30.3 | 1.9 | 22.2 | 0.7 | 27.1 | 0.7 | 28.3 | 0.2 | 35.8 | 0.6 | 38.8 | NA | 31.6 | 1.5 | 35.9 | 0.2 | 30.4 | 0.5 | 38.7 | 1.6 | 33.8 | 0.8 | 37.4 | 0.8 | 39.6 | NA |
| 12417 | Ru | AFM | 28.4 | 0.6 | 23.4 | 0.7 | 25.6 | 0.7 | 27.8 | NA | 37.0 | 0.0 | NA | NA | 32.7 | 0.5 | 38.2 | 1.0 | 29.5 | 1.5 | NA | NA | 21.5 | 1.3 | 38.4 | 1.4 | 36.5 | NA |
| 12421 | Nu | AFM | 27.9 | 0.7 | 22.9 | 4.0 | 25.4 | 0.5 | 28.5 | 0.1 | 33.0 | 0.7 | 35.7 | 0.1 | 30.7 | 0.5 | 36.0 | 0.2 | 30.1 | 0.2 | 38.5 | 0.1 | 30.2 | 1.2 | 38.4 | NA | 35.7 | 0.3 |
| 12422 | Nu | AFM | 28.8 | 0.2 | 21.7 | 2.1 | 25.5 | 0.3 | 27.4 | 0.0 | 33.9 | 1.2 | 38.9 | 0.1 | 30.3 | 0.5 | 36.4 | 1.0 | 30.5 | 1.1 | 35.1 | 0.7 | 33.1 | 0.7 | 35.6 | 0.8 | 37.4 | NA |
| 12426 | Nu | AFM | 28.0 | NA | 21.0 | 2.1 | 24.8 | 0.4 | 26.7 | 0.1 | 31.8 | 0.4 | NA | NA | 31.8 | 0.9 | 35.1 | 0.4 | 27.8 | 0.1 | 38.5 | NA | 26.8 | 1.4 | NA | NA | 37.3 | 0.2 |
| 12427 | Nu | AFM | 30.4 | 0.0 | 22.4 | 2.0 | 25.4 | 1.0 | 28.6 | 0.3 | 34.9 | NA | 39.6 | NA | 30.6 | 0.9 | 38.5 | NA | 30.0 | 0.0 | NA | NA | 26.6 | 0.4 | 39.2 | NA | 37.9 | 0.2 |
| 12428 | Ni | AFM | 26.4 | 1.0 | 20.1 | 1.3 | 23.0 | 0.8 | 23.4 | 0.9 | 32.4 | 0.6 | 37.5 | 1.7 | 27.9 | 0.5 | 33.9 | 1.2 | 27.9 | 0.0 | 26.9 | 0.0 | 17.6 | 0.5 | 33.2 | 0.8 | 37.7 | 0.6 |
| 12442 | Ru | AFM | 29.5 | 0.3 | 23.6 | 0.6 | 26.1 | 1.0 | 28.2 | 0.2 | 33.8 | 0.3 | 38.3 | NA | 30.4 | 0.6 | 34.6 | 1.9 | 28.6 | 0.3 | NA | NA | 24.7 | 0.1 | 37.4 | 0.2 | 37.0 | 0.4 |
| 12446 | Ri | AFM | 26.9 | 1.0 | 21.0 | 0.2 | 25.2 | 0.5 | 26.2 | 0.1 | 31.8 | 0.1 | 35.9 | 0.3 | NA | NA | NA | NA | 28.2 | 1.1 | 27.3 | 0.0 | 18.2 | 0.6 | 30.2 | 0.2 | 37.1 | 0.5 |
| 12463 | Ri | AFM | 26.9 | 1.8 | 18.9 | 0.4 | 24.6 | 0.6 | 25.7 | 0.0 | 32.1 | 0.3 | 37.0 | 1.2 | 30.4 | 1.3 | 36.1 | 0.3 | 28.8 | 0.3 | 30.8 | 0.0 | 19.0 | 0.7 | 36.1 | 0.1 | 37.3 | 0.2 |
| 12464 | Ri | AFM | 30.7 | 1.1 | 20.9 | 0.7 | 24.9 | 0.4 | 29.7 | 0.2 | 33.7 | 0.2 | 36.0 | 1.0 | 29.2 | 0.7 | 36.4 | 0.7 | 30.4 | 0.4 | 28.6 | 0.1 | 20.2 | 0.4 | 33.1 | 0.5 | 39.0 | 0.0 |
| 12468 | Nu | AFM | 27.7 | 1.4 | 20.8 | 0.6 | 24.6 | 0.6 | 28.8 | 0.2 | 34.1 | 1.0 | 37.8 | 0.8 | 29.8 | 0.8 | 34.8 | 0.6 | 28.1 | 0.0 | 38.5 | NA | 29.1 | 0.8 | 36.0 | 0.1 | 36.6 | 0.0 |
| 12469 | Ri | AFM | 26.6 | NA | 20.9 | 0.9 | 24.0 | 0.3 | 28.3 | 0.0 | 32.3 | 0.0 | 36.4 | 0.3 | 28.4 | 0.6 | 35.6 | 0.4 | 29.8 | 0.6 | 30.7 | 0.0 | 27.4 | 0.4 | 30.7 | 0.0 | 36.8 | 0.4 |
| 12470 | Ri | AFM | 28.6 | 1.0 | 22.6 | 0.5 | 25.6 | 0.7 | 28.9 | 0.1 | 33.0 | 0.9 | 36.1 | 1.2 | 26.7 | 0.4 | 34.4 | 0.1 | 29.3 | 0.5 | 29.2 | 0.5 | 28.6 | 0.2 | 31.7 | 0.0 | 36.4 | 0.8 |
| 12474 | Ni | AFM | 28.3 | 0.8 | 21.8 | 0.5 | 24.7 | 0.8 | 28.4 | 0.1 | 34.6 | 0.1 | 38.2 | 1.1 | 31.1 | 0.3 | 33.7 | 0.1 | 28.8 | 0.0 | 36.0 | 1.3 | 30.3 | 0.3 | 34.1 | 0.1 | 38.4 | 0.8 |
| 12485 | Ni | AFM | 28.7 | 0.2 | 21.0 | 0.2 | 26.1 | 0.6 | 28.2 | 0.2 | 31.7 | 0.3 | 37.3 | 1.2 | 29.8 | 0.8 | 34.4 | 0.3 | 29.1 | 0.6 | 29.6 | 0.0 | 30.4 | 0.0 | 32.2 | 0.2 | 38.6 | 0.4 |
| 12491 | Ni | AFM | 28.9 | 0.1 | 20.2 | 0.6 | 27.1 | 0.0 | 27.6 | 0.2 | 32.3 | 0.2 | 36.8 | 0.7 | 28.8 | 0.6 | 35.2 | 0.5 | 29.9 | 0.2 | 26.5 | 0.0 | 18.5 | 0.1 | 33.1 | 0.1 | 38.9 | 0.4 |
| 12492 | Ri | AFM | 28.1 | 0.0 | 20.1 | 1.0 | 26.2 | 0.8 | 26.0 | 0.2 | 31.2 | 0.5 | 36.1 | 0.7 | 27.3 | 0.4 | 35.4 | 0.3 | 29.0 | 0.2 | 24.9 | 0.2 | 18.4 | 0.0 | 32.5 | 0.0 | 38.7 | 0.2 |
| 12493 | Ri | AFM | 35.1 | 0.4 | 31.8 | 1.1 | NA | NA | 34.9 | 0.7 | 38.2 | 1.4 | 37.8 | NA | 32.4 | 0.9 | 36.9 | 0.3 | 34.2 | 1.3 | NA | NA | 32.1 | 0.0 | NA | NA | NA | NA |
| 12498 | Ni | AFM | 26.8 | 0.0 | 20.2 | 0.7 | 26.2 | 0.1 | 26.3 | 0.0 | 32.0 | 0.2 | 38.4 | 0.4 | 28.7 | 1.0 | 33.9 | 0.1 | 27.6 | 0.4 | 28.4 | 0.2 | 26.8 | 0.0 | 34.0 | 0.2 | 36.2 | 0.3 |
| 12514 | Ni | AFM | 29.6 | 1.1 | 21.9 | 0.3 | 26.7 | 0.9 | 29.0 | 0.2 | 33.4 | 0.1 | 38.7 | 0.2 | 31.7 | 1.3 | 36.8 | 0.4 | 29.7 | 0.1 | 32.4 | 0.0 | 28.7 | 0.1 | 33.1 | 0.4 | 38.2 | NA |
| 12526 | Ru | AFM | 28.8 | 0.3 | 21.7 | 0.8 | 27.5 | 0.2 | 28.5 | 0.1 | 33.7 | 0.3 | NA | NA | 32.2 | 0.7 | 38.0 | 1.4 | 31.6 | 0.1 | 40.0 | NA | 25.8 | 0.1 | NA | NA | 38.2 | 0.3 |
| 12529 | Ni | AFM | 28.2 | 0.7 | 22.4 | 0.6 | 25.1 | 0.3 | 27.8 | 0.1 | 34.0 | 0.5 | 37.8 | 1.3 | 30.7 | 0.9 | 36.6 | 0.3 | 29.9 | 0.5 | 35.4 | 0.3 | 30.4 | 0.1 | 35.7 | 0.4 | 38.4 | 0.3 |
| 12558 | Ni | AFM | 26.0 | 0.5 | 23.2 | 4.6 | 26.9 | 3.7 | 26.1 | 0.1 | 31.6 | 0.2 | 38.7 | 0.4 | 30.4 | 0.6 | 35.7 | 0.6 | 29.5 | 0.4 | 31.9 | 0.2 | 31.7 | 0.1 | 38.6 | 0.5 | 38.3 | 0.5 |
| 12559 | Ru | AFM | 27.9 | 0.7 | 22.7 | 3.7 | 27.9 | 3.9 | 28.6 | 0.1 | 34.4 | 1.0 | 38.0 | 0.0 | 30.7 | 0.2 | 35.2 | 0.1 | 28.8 | 0.5 | NA | NA | 32.3 | 0.1 | 36.1 | 0.2 | 36.9 | 0.8 |
| 12562 | Ri | AFM | 29.0 | 0.3 | 22.7 | 3.6 | 27.7 | 1.7 | 28.4 | 0.1 | 32.7 | 0.3 | 37.2 | 1.0 | 29.9 | 0.5 | 36.6 | 0.1 | 30.9 | 0.1 | 32.7 | 0.1 | 20.5 | 0.2 | 35.5 | 0.1 | 39.1 | NA |
| 12566 | Ri | AFM | 26.9 | 0.1 | 21.9 | 1.7 | 26.2 | 1.4 | 26.3 | 0.0 | 32.1 | 0.2 | 36.4 | 0.5 | 26.5 | 0.3 | 34.9 | 0.9 | 28.0 | 0.4 | 26.2 | 0.3 | 18.4 | 0.1 | 33.5 | 0.4 | 37.1 | 0.4 |

**B Geometric mean (µ) and associated standard deviation (s) of measured Ct values for each sheep x gene combination in abomasal lymph node (ALN).**

| Sheep | Group | Tissue | Housekeeping genes | | | | | | | | | | Genes of interest | | | | | | | | | | | | | | | |
| --- | --- | --- | --- | --- | --- | --- | --- | --- | --- | --- | --- | --- | --- | --- | --- | --- | --- | --- | --- | --- | --- | --- | --- | --- | --- | --- | --- | --- |
|  |  |  | HPRT | | S26Q | | SDH | | TYQ | | BACTIN | | IL4 | | IL13 | | IFNg | | TNFa | | CCL26 | | TNFRSF4 | | CXCL14 | | PAPPA2 | |
|  |  |  | m | s | m | s | m | s | m | s | m | s | m | s | m | s | m | s | m | s | m | s | m | s | m | s | m | s |
| 12402 | Ru | ALN | 26.8 | 2.2 | 26.4 | 0.2 | 29.3 | 0.2 | 24.8 | 0.2 | 34.8 | 0.0 | 35.7 | 0.5 | 30.7 | 0.8 | 36.5 | 0.4 | 28.9 | 0.1 | 32.4 | 0.4 | 29.7 | 0.0 | 33.3 | 0.2 | 37.5 | 0.5 |
| 12417 | Ru | ALN | 26.0 | 0.7 | 26.3 | 0.0 | 29.6 | 0.1 | 25.6 | 0.0 | 35.2 | 0.5 | 36.0 | 0.4 | 29.1 | 0.2 | 36.0 | 0.3 | 28.7 | 0.1 | 33.8 | 0.0 | 30.8 | 0.1 | 33.2 | NA | 36.7 | 0.4 |
| 12421 | Nu | ALN | 25.7 | 0.9 | 25.9 | 0.1 | 29.0 | 0.0 | 25.4 | 0.0 | 34.4 | 0.8 | 35.4 | 0.5 | 29.1 | 0.3 | 37.0 | 1.2 | 29.2 | 0.1 | 30.6 | 0.3 | 30.5 | 0.6 | 31.9 | 0.0 | 37.4 | 0.6 |
| 12422 | Nu | ALN | 29.4 | 0.6 | 28.4 | 0.1 | 31.7 | 0.0 | 27.5 | 0.0 | 36.7 | 0.7 | 38.6 | NA | 32.3 | 0.2 | 40.0 | NA | 30.6 | 0.0 | 34.5 | 0.1 | 32.7 | 0.4 | 35.1 | NA | 38.4 | 0.3 |
| 12426 | Nu | ALN | 27.9 | 0.7 | 27.2 | 0.4 | 30.1 | 0.1 | 26.9 | 0.1 | 36.6 | 0.1 | 36.4 | 1.4 | 29.7 | 0.2 | 36.0 | 0.3 | 28.7 | 0.0 | 32.1 | 0.1 | 30.9 | 0.7 | 33.3 | 0.1 | 37.1 | 0.4 |
| 12427 | Nu | ALN | 29.6 | 0.9 | 27.9 | 0.2 | 31.1 | 0.1 | 27.6 | 0.0 | 36.3 | 0.9 | 36.3 | 0.6 | 29.6 | 0.5 | 37.0 | 0.0 | 30.0 | 0.0 | 34.6 | 1.4 | 31.3 | 0.3 | 33.8 | 0.1 | 38.0 | 0.7 |
| 12428 | Ni | ALN | 25.2 | 0.1 | 26.2 | 0.2 | 28.6 | 0.1 | 24.2 | 0.1 | 34.5 | 0.4 | 33.6 | NA | 29.1 | 0.1 | 35.9 | 0.6 | 28.0 | 0.0 | 31.9 | 0.3 | 29.6 | 0.6 | 31.1 | 0.0 | 36.4 | 0.4 |
| 12442 | Ru | ALN | 27.2 | 1.4 | 26.6 | 0.8 | 30.3 | 0.1 | 25.6 | 0.1 | 35.8 | 0.6 | 37.0 | 0.6 | 30.5 | 0.0 | 36.1 | 0.2 | 28.8 | 0.3 | 32.5 | 1.3 | 29.5 | 0.4 | 33.5 | 0.3 | 37.7 | 0.4 |
| 12446 | Ri | ALN | 25.9 | 1.0 | 26.5 | 0.2 | 30.0 | 0.2 | 25.0 | 0.2 | 34.3 | 0.3 | 33.8 | 0.1 | 29.2 | 0.5 | 37.2 | 0.7 | 29.7 | 0.0 | 30.0 | 0.3 | 30.5 | 0.4 | 33.9 | 0.2 | 38.6 | NA |
| 12463 | Ri | ALN | 25.0 | 0.8 | 24.9 | 0.4 | 28.2 | 0.1 | 24.3 | 0.0 | 33.8 | 0.3 | 33.1 | 0.0 | 29.9 | 0.1 | 35.2 | 0.0 | 28.1 | 0.1 | 32.9 | 0.4 | 30.1 | 0.6 | 31.2 | 0.0 | 37.0 | 0.1 |
| 12464 | Ri | ALN | 27.5 | 0.7 | 26.4 | 0.5 | 29.9 | 0.0 | 26.3 | 0.0 | 34.9 | NA | 35.3 | 0.4 | 29.8 | 0.3 | 36.9 | 0.7 | 28.9 | 0.0 | 31.2 | 0.2 | 30.8 | 0.3 | 33.7 | 0.2 | 38.0 | 0.0 |
| 12468 | Nu | ALN | 28.3 | 0.9 | 27.2 | 0.2 | 30.4 | 0.4 | 27.0 | 0.1 | 35.8 | 0.3 | 35.7 | 0.2 | 28.9 | 0.4 | 37.2 | 0.2 | 29.4 | 0.1 | 32.8 | 0.5 | 31.2 | 0.2 | 33.7 | 0.4 | 38.0 | 0.1 |
| 12469 | Ri | ALN | 24.4 | 0.9 | 25.8 | 0.2 | 28.7 | 0.0 | 24.3 | 0.0 | 33.1 | 0.3 | 32.6 | 0.3 | 29.3 | 0.2 | 37.0 | 0.8 | 28.3 | 0.1 | 32.5 | 0.6 | 30.2 | 0.2 | 32.2 | 0.1 | 37.2 | 0.5 |
| 12470 | Ri | ALN | 24.5 | 1.0 | 25.9 | 0.1 | 30.3 | 0.0 | 24.9 | 0.1 | 32.5 | 0.2 | 33.3 | 0.3 | 28.5 | 0.3 | 36.9 | 0.1 | 27.7 | 0.1 | 32.4 | 0.5 | 30.2 | 0.3 | 32.2 | 0.2 | 36.3 | 0.2 |
| 12474 | Ni | ALN | 26.1 | 0.9 | 27.3 | 0.0 | 29.6 | 0.0 | 25.7 | 0.1 | 34.3 | 0.2 | 35.7 | 0.5 | 28.9 | 0.0 | 37.4 | 0.2 | 28.9 | 0.1 | 35.9 | NA | 29.8 | 0.1 | 33.3 | 0.1 | 37.1 | 0.2 |
| 12485 | Ni | ALN | 26.0 | 1.0 | 25.9 | 1.2 | 28.7 | 0.1 | 24.6 | 0.1 | 34.0 | 0.2 | 33.6 | 0.3 | 27.8 | 0.2 | 36.2 | 0.5 | 27.7 | 0.1 | 29.3 | 0.2 | 30.1 | 0.6 | 32.5 | 0.1 | 37.0 | 0.4 |
| 12491 | Ni | ALN | 25.2 | 0.6 | 26.4 | 0.7 | 29.5 | 0.0 | 24.6 | 0.0 | 34.4 | 0.3 | 34.7 | 0.0 | 30.9 | 0.1 | 35.4 | 0.0 | 27.6 | 0.1 | 31.7 | 0.4 | 29.4 | 2.3 | 31.1 | 0.0 | 36.9 | 0.2 |
| 12492 | Ri | ALN | 24.3 | 0.8 | 26.1 | 1.0 | 29.5 | 0.1 | 23.9 | 0.0 | 33.5 | 0.1 | 34.3 | 0.1 | 30.3 | 0.5 | 36.3 | 0.4 | 28.2 | 0.1 | 30.9 | 0.1 | 29.5 | 0.2 | 31.6 | 0.1 | 36.6 | 0.1 |
| 12493 | Ri | ALN | 25.0 | 0.6 | 26.3 | 0.5 | 29.0 | 0.0 | 24.4 | 0.1 | 34.6 | 0.4 | 33.7 | 0.0 | 29.9 | 0.2 | 37.6 | 0.4 | 28.2 | 0.0 | 30.0 | 0.1 | 30.1 | 0.9 | 31.2 | 0.2 | 36.5 | 0.1 |
| 12498 | Ni | ALN | 25.0 | 0.9 | 26.6 | 0.1 | 29.4 | 0.1 | 24.5 | 0.0 | 34.9 | 0.5 | 33.4 | 0.1 | 29.2 | 0.5 | 36.6 | 0.4 | 28.3 | 0.0 | 31.0 | 0.3 | 30.8 | 0.7 | 32.2 | 0.0 | 38.4 | 0.1 |
| 12514 | Ni | ALN | 24.3 | 0.8 | 26.2 | 0.0 | 28.2 | 0.1 | 23.6 | 0.0 | 34.1 | 0.0 | 32.6 | 0.0 | 30.0 | 0.5 | 37.3 | 0.4 | 28.2 | 0.1 | 31.0 | 0.1 | 29.8 | 1.1 | 30.8 | 0.1 | 36.8 | 0.1 |
| 12526 | Ru | ALN | 26.7 | 1.4 | 26.5 | 0.3 | 29.9 | 0.0 | 26.3 | 0.1 | 35.8 | 0.2 | 35.4 | 1.0 | 29.2 | 0.4 | 35.4 | 0.2 | 28.8 | 0.3 | 30.8 | 0.1 | 30.3 | 0.4 | 33.0 | 0.1 | 37.6 | 0.4 |
| 12529 | Ni | ALN | 25.3 | 0.9 | 26.7 | 0.9 | 28.8 | 0.0 | 23.8 | 0.1 | 33.8 | 0.3 | 34.7 | 0.5 | 30.2 | NA | 36.8 | 0.0 | 28.5 | 0.1 | 30.8 | 0.0 | 29.8 | 0.1 | 31.0 | 0.1 | 36.6 | 0.0 |
| 12558 | Ni | ALN | 26.0 | 1.0 | 26.4 | 0.3 | 30.0 | 0.1 | 24.8 | 0.4 | 35.2 | 0.4 | 33.4 | 0.2 | 30.9 | 0.1 | 36.3 | 0.1 | 28.4 | 0.1 | 33.4 | 0.0 | 30.1 | 0.8 | 32.9 | 0.2 | 38.6 | 0.9 |
| 12559 | Ru | ALN | 31.8 | 0.6 | 28.5 | 0.1 | 33.1 | 0.3 | 28.6 | 0.0 | 38.5 | 0.1 | 38.2 | NA | 30.2 | 0.1 | 40.0 | NA | 30.2 | 0.2 | 34.3 | 0.6 | 33.8 | 0.2 | 39.0 | NA | 38.9 | NA |
| 12562 | Ri | ALN | 28.0 | 0.1 | 26.7 | 0.1 | 30.0 | 0.0 | 26.1 | 0.1 | 36.0 | 0.0 | 36.0 | 0.2 | 30.7 | 0.3 | 38.5 | 0.5 | 29.7 | 0.0 | 33.4 | 1.2 | 31.2 | 0.4 | 33.9 | 0.2 | 38.7 | 0.7 |
| 12566 | Ri | ALN | 25.0 | 1.6 | 25.8 | 0.2 | 28.5 | 0.1 | 23.8 | 0.1 | 34.3 | 0.1 | 33.3 | 0.2 | 29.2 | 0.2 | 36.3 | 0.5 | 27.6 | 0.3 | 28.5 | 0.0 | 30.0 | 0.6 | 31.1 | 0.0 | 36.1 | 0.2 |
