# Supplementary material for: Functional investigation of a QTL affecting resistance to Haemonchus contortus in sheep
Source: Vet Res. 2014 Jun 17;45(1):68. doi: 10.1186/1297-9716-45-68 (PMC4077151; doi:10.1186/1297-9716-45-68)
Supplement: Additional file 4 — List of parasitological and haematological traits in the R and N sheep. The average performance of the R and N sheep is provided for every single trait monitored during experimental challenge by H. contortus and after necropsy. Average raw data are provided for the ease of reading, while statistical tests were performed after correction for fixed effect (e.g. sheep sex) and statistical transformation when appropriate. [file 1297-9716-45-68-S4.doc]

## Additional file 4 – List of parasitological and haematological traits in the R and N groups of BCxBC sheep

| Trait | R | | S | | *p*-value |
| --- | --- | --- | --- | --- | --- |
|  | Mean | Std | Mean | Std |  |
| FEC18 (eggs/g) | 126 | 289 | 727 | 1153 | 0.0215 |
| FEC21(eggs/g) | 1160 | 1813 | 3865 | 3705 | 0.0646 |
| FEC24 (eggs/g) | 3874 | 3031 | 6968 | 5233 | 0.1909 |
| FEC27 (eggs/g) | 5798 | 4075 | 9150 | 6422 | 0.1118 |
| FEC30 (eggs/g) | 13213 | 8286 | 24759 | 17730 | 0.0147 |
| WB (no. Worms) | 4084 | 1885 | 4395 | 1267 | 0.7345 |
| FL (mm) | 18.7 | 1.4 | 20.3 | 1.6 | 0.0013 |
| FF (no. Eggs in utero) | 358 | 112 | 525 | 182 | 0.0005 |
| HCT0 (%) | 36 | 3 | 36 | 4 | 0.6233 |
| HCT14 (%) | 34 | 3 | 32 | 4 | 0.0178 |
| HCT27 (%) | 30 | 3 | 27 | 3 | 0.0129 |
| MCHC0 (g/dL) | 34 | 2 | 34 | 1 | 0.6536 |
| MCHC14 (g/dL) | 34 | 2 | 34 | 1 | 0.5802 |
| MCHC27 (g/dL) | 31 | 2 | 31 | 2 | 0.4634 |
| LYMPH0 (10^3^/ µL) | 6.29 | 1.36 | 5.29 | 0.80 | 0.01 |
| LYMPH14 (10^3^/ µL) | 5.67 | 1.24 | 5.00 | 0.87 | 0.8055 |
| LYMPH27 (10^3^/ µL) | 4.40 | 1.53 | 4.09 | 0.75 | 0.7024 |
| MONO0 (10^3^/ µL) | 0.70 | 0.29 | 0.73 | 0.38 | 0.8283 |
| MONO14 (10^3^/ µL) | 0.69 | 0.24 | 0.70 | 0.32 | 0.7819 |
| MONO27 (10^3^/ µL) | 0.73 | 0.29 | 0.68 | 0.27 | 0.3944 |
| EO0 (10^3^/ µL) | 0.09 | 0.07 | 0.13 | 0.16 | 0.4783 |
| EO14 (10^3^/ µL) | 0.14 | 0.07 | 0.12 | 0.10 | 0.4729 |
| EO27 (10^3^/ µL) | 0.06 | 0.03 | 0.06 | 0.07 | 0.5265 |
| BASO0 (10^3^/ µL) | 0.09 | 0.05 | 0.05 | 0.02 | 0.0058 |
| BASO14 (10^3^/ µL) | 0.06 | 0.05 | 0.04 | 0.02 | 0.5286 |
| BASO27 (10^3^/ µL) | 0.07 | 0.03 | 0.04 | 0.02 | 0.1459 |
| NEUT0 (10^3^/ µL) | 2.54 | 0.78 | 2.96 | 0.68 | 0.1285 |
| NEUT14 (10^3^/ µL) | 2.58 | 0.78 | 2.61 | 0.72 | 0.4263 |
| NEUT27 (10^3^/ µL) | 2.31 | 0.79 | 2.40 | 0.73 | 0.6527 |
| RET0 (%) | 0.06 | 0.03 | 0.06 | 0.03 | 0.2185 |
| RET14 (%) | 0.05 | 0.03 | 0.05 | 0.01 | 0.8014 |
| RET27 (%) | 0.17 | 0.20 | 0.82 | 1.01 | 0.0067 |
| PLT0 (10^3^ / µL) | 372 | 85 | 400 | 107 | 0.3315 |
| PLT14 (10^3^ / µL) | 441 | 101 | 448 | 93 | 0.7474 |
| PLT27 (10^3^ / µL) | 459 | 105 | 480 | 85 | 0.868 |
| BW1 (kg) | 39.82 | 6.78 | 38.54 | 7.64 | 0.0725 |
| BW2 (kg) | 43.08 | 7.28 | 42.64 | 8.44 | 0.1875 |
| ADG (kg/day) | 0.109 | 0.045 | 0.137 | 0.049 | 0.2287 |
